# 53BP1 suppresses tumor growth and promotes susceptibility to apoptosis of ovarian cancer cells through modulation of the Akt pathway

**DOI:** 10.3892/or.2012.1641

**Published:** 2012-01-18

**Authors:** SHUHUI HONG, XIAOYAN LI, YING ZHAO, QIFENG YANG, BEIHUA KONG

**Affiliations:** 1 Department of Obstetrics and Gynecology, Qilu Hospital, Shandong University, Ji’nan, Shandong 250012; 2 Department of Gynecology and Obstetrics, Affiliated Qianfoshan Hospital of Shandong University, Ji’nan, Shandong 250014; 3 Department of General Surgery, Qilu Hospital, Shandong University, Ji’nan, Shandong 250012, P.R. China

**Keywords:** 53BP1, proliferation, cell cycle, apoptosis, p-Akt, ovarian cancer

## Abstract

53BP1 has been extensively studied as a key component of the DNA damage response, but little is known regarding the role of 53BP1 in preventing tumor development. The present study was designed to assess the impact of the modification of 53BP1 gene expression on the biological behavior of ovarian cancer cell lines and to elucidate the cellular pathway(s) triggered by 53BP1 in cancer cells. DNA liposome transfection technology was employed to increase and to knock down the expression of 53BP1 in A2780 and HO-8910PM cells, respectively. Viability, clonogenicity and cell cycle profiles were evaluated. Cell apoptosis was analyzed using flow cytometric assay. The expression of proteins related to apoptosis and cell signal transduction was assessed using western blotting. Increased expression of 53BP1 decreased the viability and the clonogenicity, and induced G_2_/M arrest and apoptosis of the treated cells. The protein expression of Bax, P21 and caspase-3 was upregulated, while the levels of Bcl-2 and p-Akt were reduced to a statistically significant level. In contrast, deregulation of 53BP1 significantly increased proliferative ability. Collectively, our data suggest that 53BP1 is involved in several important steps in controlling cell proliferation and growth and preventing tumor development.

## Introduction

Ovarian cancer is a common aggressive disease and is the fifth leading cause of cancer-related death in women (1). The life-time risk of ovarian cancer is approximately 1.4% (2). Approximately 200,000 women are diagnosed with this highly lethal disease each year (3). Due to the lack of obvious symptoms, substantial delays in diagnosis are quite common, and the overall survival rate for ovarian cancer has only slightly increased over the last 30 years in spite of advances in surgery and chemotherapy. Therefore, understanding of the tumor molecular biology is an essential step for the selection of the most effective treatment strategy for ovarian cancer.

Tumorigenesis is a result of the accumulation of genetic changes. Genomic instability leads to the transformation of normal cells to cancer cells. This includes aberrant reduction or loss of tumor-suppressor genes and activation of proto-oncogenes (4).

P53 binding-proteιn 1 (53BP1) was initially identified in a yeast two-hybrid system by Iwabuchi *et al* in 1994, and has been mapped to chromosomes 15q15–21 and predicts a protein of 1972 amino acids (217 kb) (5). Similar to other mediators of checkpoint responses (e.g., BRCA1, MDC1), 53BP1 also contains a highly conserved tandem BRCA1 C-terminal (BRCT) domain, which is essential for tumor-suppressor function via interaction with ezrin-radixin-moesin (ERM) at the plasma membrane (6). Owing to its rapid accumulation to sites of DNA double-strand breaks (DSB), 53BP1 has been considered a cytologic marker for endogenous DSB (7). The functions of 53BP1 range from participation in DNA-damage repair to negotiating cell cycle checkpoints (7,8). By triggering a cell cycle checkpoint, cells suffering DNA damage undergo cell cycle arrest and apoptosis avoiding genomic instability which can generate cancer (9). Defects in 53BP1 were found to contribute to the pathogenesis of various types of human cancer (10,11), but our knowledge of the role of 53BP1 in ovarian cancer is still rudimentary. Here, we presented a preliminary analysis of 53BP1 in the prevention of ovarian cancer development.

## Materials and methods

*E. coli* DH5α competent cells, Lipofectamine 2000 and RPMI-1640 medium were purchased from Invitrogen (Carlsbad, CA, USA). N-Myc-53BP1 WT pLPC-Puro, pSUPER-neo-GFP, and pSUPER-neo-GFP-53BP1 were purchased from Addgene, OligoEngine and Sangon Biotech (Shanghai, China), respectively. The N-Myc-WT pLPC-Puro was a kind gift from Professor Titia de Lange (Laboratory of Cell Biology and Genetics of Rockefeller University). ECL for western blotting and the gel electrophoresis device were from GE Healthcare (USA). The antibodies for Akt, p-Akt, P21^waf1/Cip1^, Bcl-2, caspase-3 and β-actin were from Cell Signaling Technologies (Boston, MA).

### Cell culture and transfection

Human ovarian cancer cell line A2780 was originally obtained from the American Type Culture Collection, and HO-8910PM, a highly metastatic ovarian cancer cell line (12), was obtained from the Chinese Academy of Sciences (Shanghai, China). The cells were grown in Rossman-Park-Memorial-Institute (RPMI)-1640 media supplemented with 10% fetal bovine serum (FBS; Haoyang Biological Manufacture Co. Ltd., Tianjin, China) at 37°C in a humidified atmosphere containing 5% CO_2_. Cells in a logarithmic growth phase were harvested for the experiments. Endogenous 53BP1 was knocked down and overexpressed using the pSUPER-neo-GFP-53BP1 and N-Myc-WT pLPC-Puro plasmids, respectively. The cells were transfected with plasmids using Lipofectamine 2000 according to the manufacturer’s protocol. Cells were selected in complete medium containing 375 μg/ml of G418 or puromycin (0.75 μg/ml) (Invitrogen). Western blotting was used to detect the expression level of 53BP1 in all of the cells described above.

### MTT assay for cell growth

The proliferative ability of cells modified by the variable gene was measured using 3-[4,5-dimethylthiazol-2-yl]-2,5-diphenyltetrazolium bromide (MTT) assay according to a standard protocol. The cells were seeded into 96-well plates (1.5×10^3^ cells/well) for 1–7 days in triplicate. At specified time points, a total of 20 μl of MTT (Amresco, Solon, OH, USA) solution (0.5 mg/ml) was added to each well, and the wells were incubated for an additional 4 h at 37°C. The purple-blue MTT formazan precipitate was dissolved in 200 μl of DMSO (Shenggong, Shanghai, China). The activity of the mitochondria, reflecting cellular growth and viability was evaluated at 550 nm with a microplate reader (Bio-Rad, Hercules, CA, USA).

### Colony forming assay

Cells (300) modified by the variable gene were plated on 60-mm culture dishes in triplicate. After cells were cultured for 10 days, colonies were fixed with methanol for 15 min and stained with 0.1% crystal violet. Colonies of at least 50 cells were counted and recorded.

### Flow cytometric analysis of the cell cycle

The transfected cells were seeded at a density of 10^6^ cells/60-mm dish (BD Biosciences, San Jose, CA, USA) in culture media contained corresponding selective antibiotics for 48 h. The cells were then washed with ice-PBS, harvested and resuspended in 1 ml staining solution (50 μg/ml PI, 20 μg/ml RNase A). After incubation of 30 min at room temperature, samples were analyzed using a FACSCalibur flow cytometer (BD Biosciences).

### Flow cytometric analysis of apoptosis

Annexin V and propidium iodide (PI) staining was performed using an Annexin V-fluorescein isothiocyanate (FITC) apoptosis detection kit (BD Biosciences) to measure apoptosis. Cells (1×10^5^) cultured in 60-mm dishes were washed twice with ice-PBS, collected, and re-suspended in 100 μl 1X binding buffer. Annexin V-FITC (5 μl) conjugate and 5 μl of PI buffer were added, and the cells were incubated at room temperature for 15 min in the dark. After addition of 400 μl of 1X binding buffer, the cells were analyzed using a flow cytometer (PE and 7AAD were used for 53BP1-knockdown HO-8910PM cells).

### Western blot analysis

Whole-cell lysates of the cells were prepared by incubating cells in RIPA buffer (Shennengbocai, Shanghai, China) (1% NP-40, 0.1% SDS, 5 mM EDTA, 0.5% sodium deoxycholate, 1 mM sodium vandate) containing protease inhibitors (1 mM PMSF, 1 mM sodium fluoride). Cell lysates were centrifuged at 12,000 rpm for 15 min at 4°C. The supernatant was collected, and the protein concentration was measured using the BCA protein assay kit (Merck, Darmstadt, Germany). Proteins in equal amounts were separated by appropriate concentration using SDS-polyacrylamide gel electrophoresis and transferred onto PVDF membranes (Millipore, Billeriaca, MA, USA). The membranes were blocked in TBST for 1 h at room temperature and then incubated with primary antibodies overnight at 4°C. The membranes were then washed three times with TBST, followed by incubation with HRP-labeled secondary antibodies (KPL, Gaithersburg, MD, USA) (1:5000). Bound antibody was visualized using ECL detection reagent (Merck).

### Statistical analysis

The SPSS version 14.0 software was used for statistical analysis. Results were reported as means ± standard deviation (SD). The Q-test was used to analyze statistical differences between groups. The alpha (α) level was set at 0.05.

## Results

### Evaluation of 53BP1 protein expression in ovarian cancer cells after transfection with the 53BP1-overpressing and -knockdown plasmids

**Figure 1:**
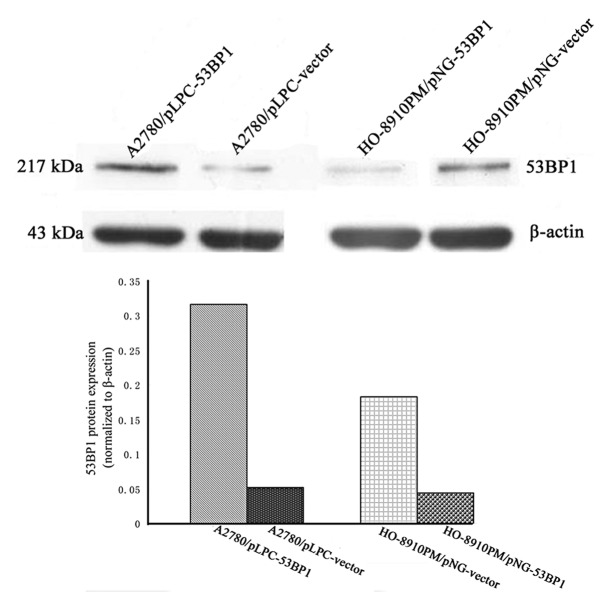
Expression level of 53BP1 in A2780/pLPC-53BP1 and HO-8910PM/pNG-vector cells was significantly increased compared with A2780/pLPC-vector and HO-8910PM/pNG-53BP1 cells, respectively. Values represent the mean ratios normalized to β-actin of three independent experiments.

Stable transfected cell lines were generated by expanding the resistant (G418 and puromycin, respectively) colonies, including A2780/pLPC-vector, A2780/pLPC-53BP1 and HO-8910PM/pNG-vector-transfected cells lines, and the expression of 53BP1 in these cell lines was examined by western blot analysis. As shown in Fig. 1, the level of 53BP1 was significantly increased and decreased compared with the corresponding control group.

### 53BP1 ectopic expression markedly alters cell proliferation in the derived cell lines of A2780 and HO-8910PM

**Figure 2:**
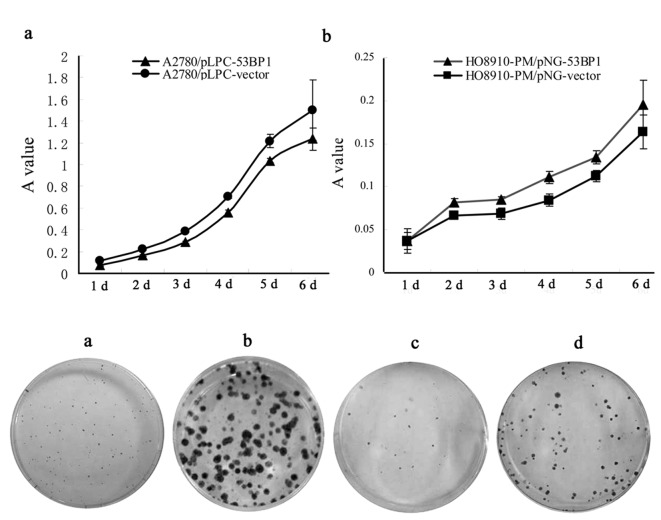
Inhibition of cell proliferation by 53BP1. The proliferation rate and the colony formation of A2780/pLPC-53BP1(Aa and Ba) and HO-8910PM/pNG-vector cells (Ab and Bc) was signifiantly reduced compared with A2780/pLPC-vector (Aa and Bb) and HO-8910PM/pNG-53BP1 cells (Ab and Bd), respectively (P<0.05).

Using MTT, we investigated the effect of 53BP1 on the proliferation of the A2780/pLPC-53BP1 cells, compared to the A2780/pLPC-vector cells, and in the HO-8910PM/pNG-53BP1 cells compared to the HO-8910PM/pNG-vector cells. As shown in Fig. 2A, A2780/pLPC-53BP1 and HO-8910PM/pNG-vector cells consistently exhibited a significant decrease in celluar proliferation compared to the A2780/pLPC-vector and HO-8910PM/pNG-53BP1 cells, respectively (P<0.05).

Colony formation assay was performed on A2780 and HO-8910PM cells, respectively. As far as congenetic cells, the colony formation significantly increased with expression of 53BP1 and vice versa (Fig. 2B).

### 53BP1-mediated Akt activation contributes to induce G_2_/M cell cycle arrest and apoptosis

**Figure 3:**
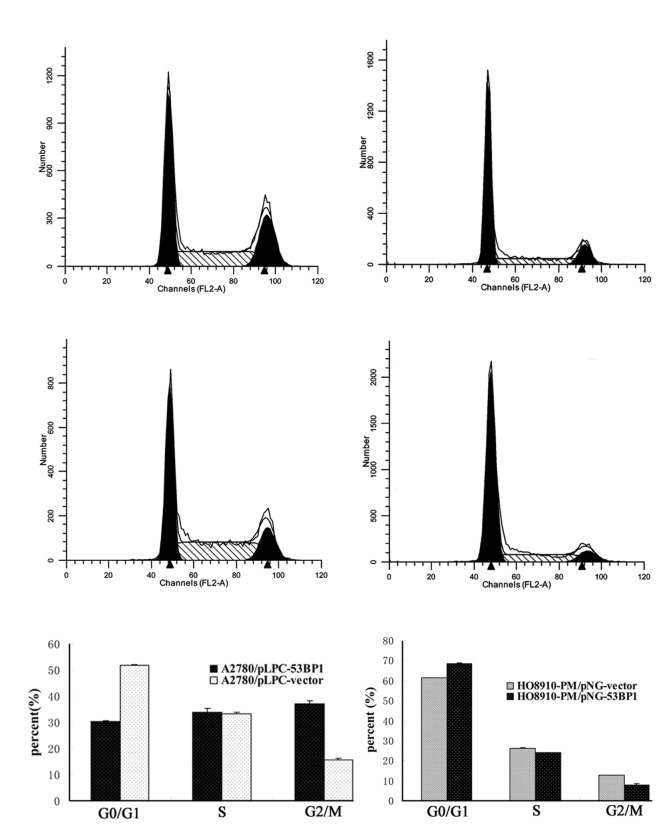
53BP1 alters the cell cycle in ovarian cancer cell lines. To explore the potential mechanism through which 53BP1 inhibits cell growth, the cell cycle was assayed by flow cytometry. As shown, compared to A2780/pLPC-vector (B, 14.93±0.58%), HO-8910PM/pNG-53BP1 (D, 7.82±0.48%), 35.90±1.30% of A2780/pLPC-53BP1 (A) and 12.52±0.32% of HO-8910PM/pNG-vector cells (C) were in G_2_/M phase. (E and F) The flow cytometric data. Data are representative of three independent experiments.

As compared to the empty vector, 53BP1 transfection caused a highly dramatical cell cycle arrest in the G_2_/M phase in A2780/pLPC-53BP1 cells (P<0.05). Similar results were found in the HO-8910PM/pNG-vector and HO-8910PM/pNG-53BP1 (P<0.05) (Fig. 3).

**Figure 4:**
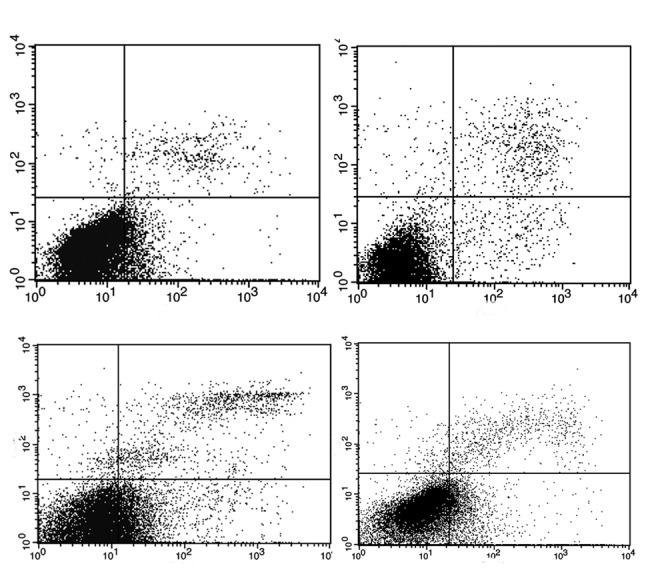
Effects of 53BP1 on the apoptosis of ovarian cancer cells. Cells were stained with Annexin V-PI, and the percentage of apoptotic cells was measured by FACSCalibur flow cytometer analysis. As shown, compared to A2780/pLPC-vector (A, 7.89±0.23%), HO-8910PM/pNG-53BP1 (C, 4.24±0.25%), 20.01±1.10% of A2780/pLPC-53BP1 (B) and 10.15±1.1% of HO-8910PM/pNG-vector cells (D) underwent apoptosis. Data are mean of three independent experiments.

Fig. 4 shows the results of Annexin V/PI staining, which was used to quantify apoptosis. The proportion of apoptotic cells in A2780/pLPC-53BP1 and A2780/pLPC-vector was ~20.01±1.10% and 7.89±0.23% (P<0.05), respectively. Downregulation of 53BP1 in the HO-8910PM/pNG-53BP1 cells decreased the proportion of apoptosis (4.24±0.25%) compared to that of the HO-8910PM/pNG-vector cells (10.15±1.1%, P<0.05)

**Figure 5:**
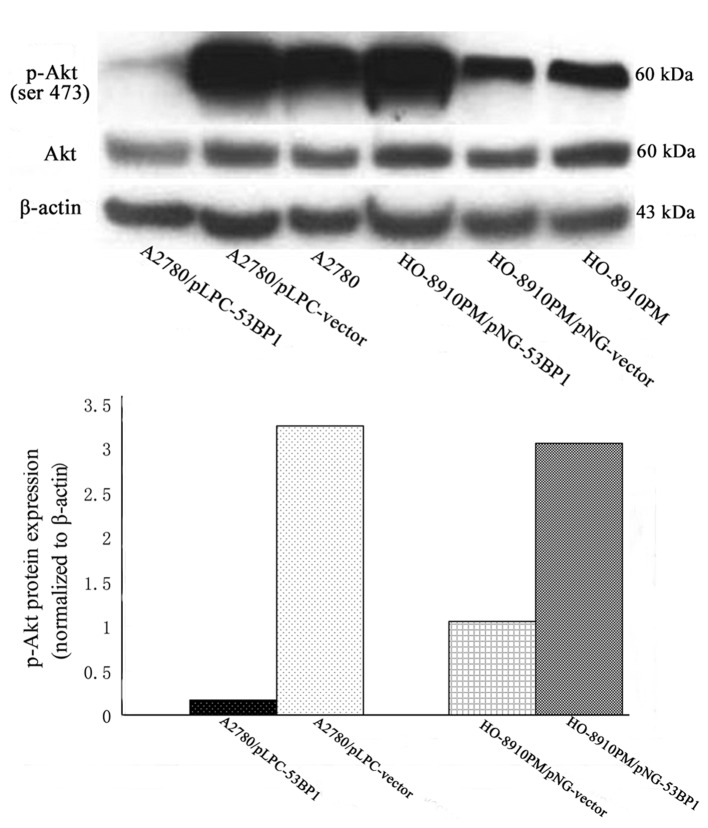
(A) Protein extracts were used for western blot analysis of Akt, p-Akt and β-actin. (B) Values represent the mean ratios normalized to β-actin for three independent experiments.

**Figure 6:**
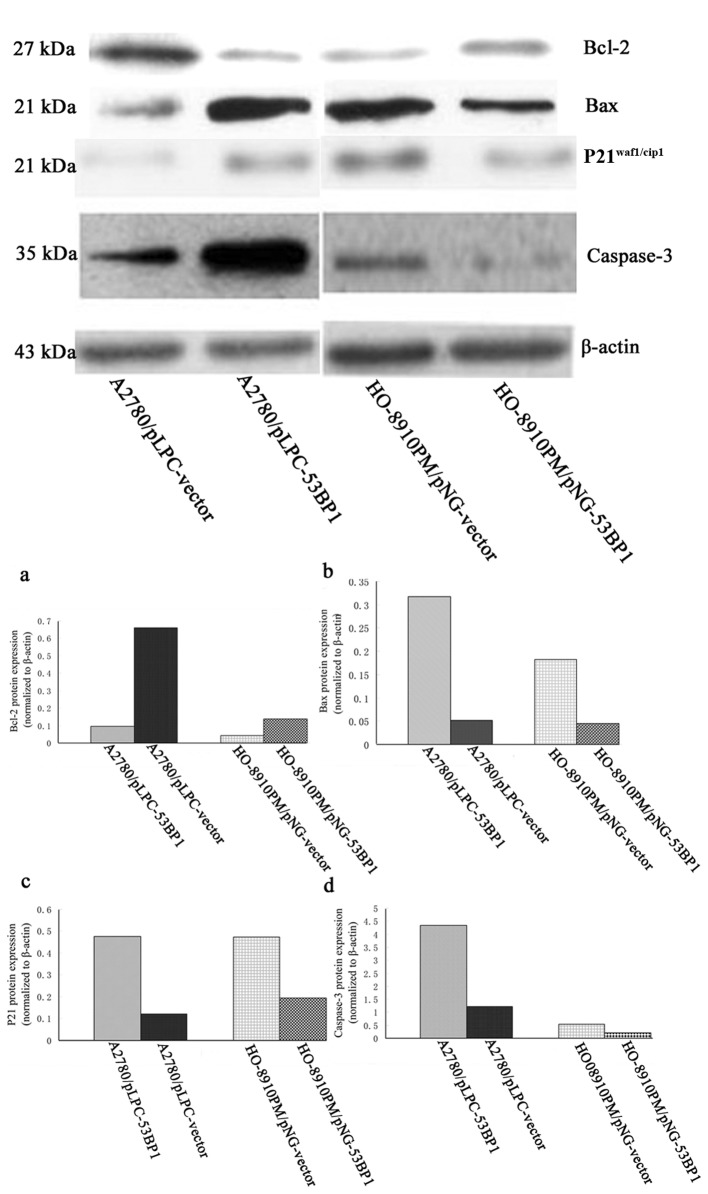
(A) Western blot assay of P21^waf1/Cip1^, caspase-3, Bax and Bcl-2 protein levels in the three cell lines. (B) Semi-quantitative analysis of (a) Bcl-2, (b) Bax, (c) P21^waf1/Cip1^ and (d) caspase-3. The protein levels were normalized to β-actin and compared with the control of three independent experiments.

When the expression of 53BP1 was relatively high, genes involved in G_2_/M phase transition and inhibition of apoptosis such as P21^waf1/Cip1^, caspase-3 and Bax were significantly upregulated (P<0.05), while, genes promoting the ability of proliferation and apoptosis, such as p-Akt, Bcl-2 were downregulated (P<0.05) (Figs. 5 and 6).

## Discussion

A healthy DNA-damage repair system is essential for maintaining a harmonious stable relationship among apoptosis, cell cycle checkpoints and cell proliferation. In contrast, a dysfunctional DNA-damage repair system results in serious consequence to cells: unlimited growth, resistance to apoptosis, and tumor formation. The crucial roles of 53BP1 in DNA-damage repair have been intensely debated. Studies have demonstrated that mutation or loss of 53BP1 is linked to a variety of common human cancers, including breast, lymphoma, and prostate cancer (11,13–15). 53BP1^−/ −^ or 53BP1^+/−^ greatly promotes lymphoma-genesis in a p53-null background (16). This present study provides a new paradigm for understanding the multiple signaling pathways involved in 53BP1 as a candidate tumor-suppressor gene which regulates ovarian cancer cell survival.

To determine whether 53BP1 plays a role in the reduction of the proliferation rate of cells, we analyzed the effect of the alteration of 53BP1 expression on the induction of antiproliferation of ovarian cancer cells, A2780 and HO-8910PM. We showed that the expression level of 53BP1 was inversely correlated to the proliferation rate of ovarian cancer cells. This effect was mostly based on the 53BP1-mediated downregulation of p-Akt (phosphorylation-Akt), while, Akt protein levels exhibited no significant change pre-and post-alteration of 53BP1. Akt is a serine/threonine protein kinase and is predominantly localized to the endoplasm (17). PIP3 (phosphatidylinositol 3,4,5-trisphosphate) synthesis is a result of activation of PI3K (PI3-kinase) by growth factor stimulation of cells. Subsequently, PIP3 triggers PDK1 (3-phosphoinositide-dependent kinase 1) to phosphorylate Akt (18). Several lines of evidence suggest that activated p-Akt serves as a multifunctional regulator of cellular processes, including cell proliferation, cell cycle and apoptosis (19–21). Research has shown that modulation of the genetic background of cancer cells or therapeutic interventions may induce G_2_/M arrest accompanied by the downregulation of p-Akt (22–24).

There is a subtle relationship among growth, apoptosis and cell cycle arrest of cancer cells. Cell cycle arrest and apoptosis can induce the inhibition of proliferation in cancer cells. Previous reports have revealed the influence of Akt on proliferation, apoptosis and cell cycle. Herein, we investigated the cell cycle profile, apoptosis and expression of related signaling proteins by modulating the expression of 53BP1 in ovarian cancer cells. We found that overexpression of 53BP1 significantly promoted apoptosis, induced G_2_/M arrest and vice versa. Furthermore, the protein levels of Bax, caspase-3 and P21^waf1/Cip1^ were significantly increased, acompanied by a decrease in Bcl-2.

Recent studies have shown that the PI3K/Akt pathway is involved in the regulation of Bcl-2 family proteins (25,26). The Bcl-2 family is an important apoptosis controller; Bcl-2 and Bax are members of the Bcl-2 family, and they belong to anti-apoptotic protein and pro-apoptotic protein, respectively. The ratio of Bax/Bcl-2 has a great influence on determining whether cells undergo apoptosis (27). In our research, we found that 53BP1 induced apoptosis of ovarian cancer cells by increasing pro-apoptotic Bax expression and decreasing anti-apoptotic Bcl-2 expression, leading to up-regulation of the ratio of Bax/Bcl-2. Although Akt plays an important role in the mechanism by which 53BP1 exerts its antiapoptosis effect, Akt is clearly not the only molecule involved. Other potential targets, such as caspase-3, were found to stimulate apoptosis. Our results confirmed the upregulation of caspase-3 expression in A2780/pLPC-53BP1 and HO-8910PM/pNG-vector cells compared to their control groups, A2780/pLPC-vector and HO-8910PM/pNG-53BP1, respectively. Bax/Bcl-2 may have an effect on caspase-3 by enhancing apoptosis of cancer cells by cytochrome c, APAF-1, caspase-9 and other death substrates (28,29). Yet, further studies are needed to confirm that other proteins are involved in the anti-apoptosis caspase pathway in relation to the effect of the alteration of 53BP1 in ovarian cancer cells. Coincident with our results, recent studies have shown that the downregulation of Akt has an impact on inducing G_2_/M arrest (22–24). P21^waf1/Cip1^ is an inhibitor of CDK (cyclin-dependent kinase) in mammalian cells and the transcriptional target of P53 (30). The tumor suppressor protein P21^waf1/Cip1^ serves to inhibit kinase activity and block progression through G_1_/S (31), while P21^waf1/Cip1^ is associated with the induction and maintenance of G_2_/M cell cycle arrest (32). Akt was found to lose the ability to play a negative role on the cell cycle by directly phosphorylating Thr145 and Ser146 of P21^waf1/Cip1^, and then the phosphorylated P21^waf1/Cip1^ is unable to enter the nucleus from the cytoplasm (33).

Overall, our results confirmed that 53BP1 inhibits cell proliferation and induces G_2_/M arrest and apoptosis. The antitumor effect of 53BP1 on ovarian cancer cells possibly involves the down-regulation of the Akt signaling pathway. These findings suggest a new target for tumors refractory to current treatments.
